# Trigger Criteria to Increase Appropriate Palliative Care Consultation in the Neonatal Intensive Care Unit

**DOI:** 10.1097/pq9.0000000000000129

**Published:** 2019-02-07

**Authors:** Lisa Humphrey, Amy Schlegel, Ruth Seabrook, Richard McClead

**Affiliations:** From the Department of Pediatrics, Nationwide Children’s Hospital, Columbus, Ohio.

## Abstract

**Introduction:**

Pediatric palliative care (PPC) seeks longitudinal relationships with patients facing life-threatening conditions. The majority of pediatric deaths occur within the first year of life, especially neonatal intensive care unit (NICU); however, the consultation by PPC in the NICU is not routine. This project sought to improve the PPC’s presence within 1 NICU for patients facing life-limiting conditions through quality improvement techniques.

**Methods:**

A trigger list of severe, life-threatening conditions impacting neonates was created and implemented to increase PPC consultation within the NICU. Interventions to improve compliance with the trigger list included the collaborative creation of the trigger list, education, modification of PPC staff modeling, and expansion of the perinatal palliative care program.

**Results:**

Over the 2 years that the project occurred, 31 prenatal and postnatal patients were eligible for PPC consultation based on the trigger list. Of these, 24 received PPC consultation. The primary outcome measure of the project was to increase PPC consultations for those NICU infants identified on a severe diagnosis “trigger” list from 25% to 80% and to maintain this increase for 6 months. This project achieved 100% compliance within 12 months.

**Conclusions:**

Utilization of quality improvement methodology to address PPC underutilization within an NICU successfully led to the implementation of a trigger list for patients with severe diagnoses to receive PPC services. Such modeling could be used in other health systems to improve palliative care referrals.

## Abstract 2

Supplemental Digital Content is available in the text.

## INTRODUCTION

In 2013, 42,328 pediatric patients died in the United States. Of these children, 55% died before the age of 1, 66% of those patients did not live past their first month, and many die in the hospital.^1^ Thus, the need for pediatric palliative care (PPC) in the neonatal intensive care unit (NICU) is significant. The American Academy of Pediatrics promotes the early and integrated involvement of PPC for families of children facing life-threatening conditions.^2^ Early introduction and long-term partnership with PPC can optimize a family-centered care approach and ensure that the care plan aligns with the family’s voiced goals of care. Unfortunately, PPC involvement in neonatal end-of-life (EOL) care remains underutilized. A review of the Pediatric Health Information System (Children’s Hospital Association, Washington, DC) database^3^ revealed that from 2001 to 2011, NICU deaths comprised 41% of all inpatient deaths; however, only 2% of those vulnerable infants and families received PPC.^4^

Our NICU also underutilized PPC. Thus, we initiated a quality improvement (QI) project for which the primary aim was to increase PPC consultation in the NICU.

## METHODS

### Setting

Nationwide Children’s Hospital (NCH) is a large, free-standing pediatric hospital in Columbus, OH, with 114 NICU beds. The hospital’s consultative PPC program comprised physicians, nurse practitioners, chaplain, and social worker. The palliative care program started in 2007. It focused on EOL care and assistance with discharge to home hospice. Hospital-wide PPC consultations averaged 5 per month. In 2013, to better meet the full definition of palliative care, the PPC team sought to expand its scope to include patients pursuing curative or life-prolonging therapies while facing life-threatening diagnoses.

#### QI Methodology

**Fig. 1.**
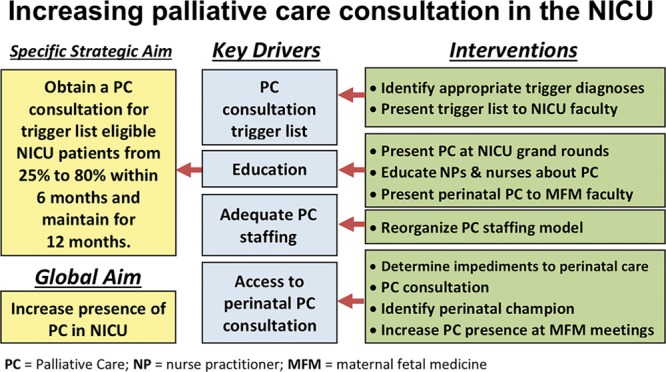
Key driver diagram for pediatric palliative care consult compliance project. MFM, maternal–fetal medicine; NP, nurse practitioner; PC, palliative care.

We established a multidisciplinary QI team and initiated a project focused on improving the PPC presence in the NICU for infants facing both EOL care and chronic, life-threatening conditions. The team included 3 neonatologists, a palliative care nurse practitioner, and a palliative care physician. We used the model for improvement and iterative interventions. The QI team reviewed current impediments and identified opportunities to improve PPC’s presence in the NICU. The group met monthly. Our specific aim was to increase PPC in the NICU for candidate patients from a baseline of 25% to 80% within 6 months and sustain for 6 months. The team identified several key drivers: (1) lack of consensus regarding which diagnoses made a patient PPC eligible, (2) NICU misperceptions and misgivings surrounding PPC, (3) insufficient PPC personnel, and (4) deficient access to perinatal palliative care (Fig. 1). As a QI project, it was not deemed human subject research; therefore, review by the NCH Institutional Review Board was not required.

### Inclusion/Exclusion Criteria

For this project, we established an initial list of chronic, life-threatening diagnoses that warranted EOL care (Table 1). We derived the list from chart query of International Classification of Diseases (ICD) codes of patients with prolonged hospitalizations that resulted in death or home hospice. This list was reviewed and revised through the expert opinion of the QI team members. These diagnoses required high decision-making complexity that would warrant PPC consultation. We considered any neonate with a diagnosis on this trigger list eligible for PPC consultation and inclusion in the project. We excluded infants without a trigger list diagnosis.

**Table 1.**
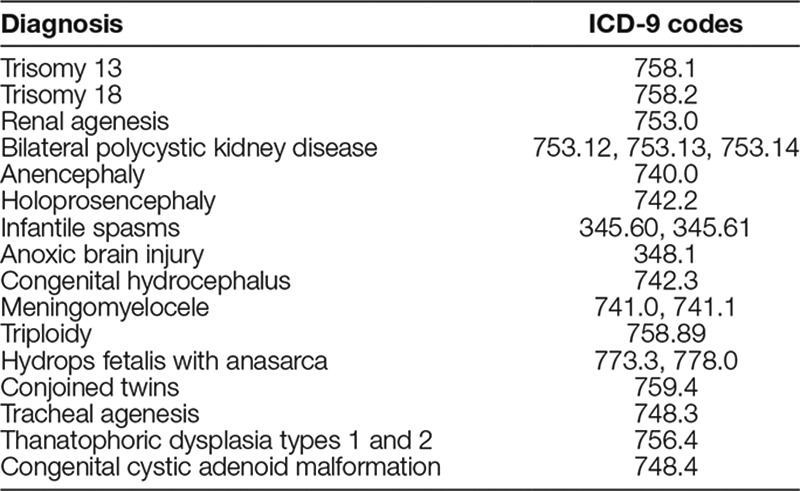
Initial List of Severe Diagnoses to Trigger Pediatric Palliative Care in the Neonatal Intensive Care Unit

### Interventions

To impact the key drivers, the QI implemented several interventions. These interventions included the following:

1. Refinement of the trigger diagnosis list to prompt PPC services: Project initiation officially started Q1 2014. For 5 monthly cycles, QI team members took turns performing manual chart reviews to validate the patient’s inclusion in the project by asking the following questions: Did the infant possess one of the trigger diagnoses? Did the infant receive a palliative care consult? Did the infant die, receive home-based palliative care/hospice, or neither? The latter was to assess if the correct level of acuity was present in the trigger list to warrant palliative care consultation. Some diagnostic codes did lack a level of acuity to warrant PPC consultation despite it being on the initial trigger list. Thus, the list was refined after 5 cycles to the 5 diagnoses that most often resulted in death or hospice referral (Table 2). The neonatology members of the QI team presented the refined trigger list of diagnoses to their NICU faculty colleagues for their concurrence that these diagnoses warranted PPC consultation.
2. Resolving misperceptions and misgivings among NICU faculty: To determine reasons for reticence in engaging PPC in the NICU, we queried the neonatologists through a focus group. Responses centered on 4 themes: (1) inaccurate definition of palliative care, (2) ignorance regarding the breadth of palliative care interventions, (3) misgivings about “needlessly upsetting parents prematurely,” and (4) belief that PPC should only be consulted if parents actively asked for hospice and verbally permitted PPC consultation. In response to these issues, the QI team neonatologists presented a lecture discussing the many interventions PPC can provide. This intervention dispelled faculty concerns that PPC consultation worsened parental angst and highlighted the ability and indications for PPC to meet even with hesitant parents.
3. Identification of a primary PPC provider for the NICU: The QI team identified inadequate staffing of the PPC team as another key driver. The lack of PPC staff as a consistent presence within the NICU significantly compromised PPC services available to families in need. Although the PPC program did not increase staffing, it reconfigured its staffing model to provide an advanced practice nurse to focus daily on NICU consultations. Her daily workflow included emphasizing PPC referral criteria, making patient rounds, attending NICU care conferences, and coordinating care.
4. Perinatal palliative care program expansion: Expert opinion on the QI team believed that PPC consultation for families who receive prenatal diagnoses of potentially life-limiting neonatal conditions ideally occurs during the prenatal period. Such timing maximally allows family members to build rapport with the PPC team and consider a full range of care plans. At the beginning of this project, a limited perinatal palliative care program existed only for families who prenatally chose no intervention at birth. We recognized the need for more comprehensive perinatal palliative care to establish early introductions, to complete family-centered advanced-care planning surrounding delivery, and to educate families on resources such as hospice or inpatient PPC. Thus, in the fourth quarter of 2014, efforts focused on expanding the perinatal palliative care program to increase prenatal referral rates, including those for trigger-listed diagnoses. Interventions included educating referring physicians, working with our prenatal liaison team to create perinatal palliative care brochures, and establishing a workflow to identify, meet, and follow these patients.

**Table 2.**
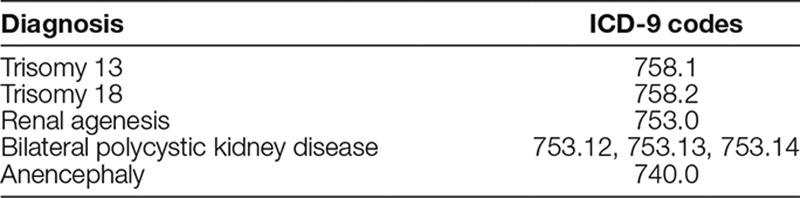
Revised List of Severe Diagnoses to Trigger Pediatric Palliative Care in the Neonatal Intensive Care Unit

### Study of the Intervention

Before project initiation, we obtained baseline NICU PPC consultation use rates for the trigger-listed diagnoses through manual chart review. The team received monthly lists of patients eligible based on ICD-9 codes for Q3 2013 and manually reviewed who received PPC consultation to establish our baseline. During this period, 4 patients were eligible, and only 1 received PPC consultation for a baseline of 25%. To assess the influence of each intervention, we measured and compared NICU physician compliance with the trigger list recommendations for PPC consultation at baseline with the postintervention rate. Interventions included education of faculty on PPC principles and the NICU trigger list, identification of primary PPC for the NICU, and expansion of the perinatal palliative care program.

### Measures and Analysis

The outcome measure of this study was the percent of eligible patients with a diagnosis on the revised trigger list (Table 2) who received a PPC consultation. Because these diagnoses were relatively rare, the percentage was determined quarterly and plotted on a run chart. The process measure was the number of days between admission to NICU and PPC consultation. We chose this measure with the goal of improving early access to PPC, thus providing optimal psychosocial and decision-making support. The balancing measure was staff satisfaction with PPC involvement. As the PPC program was already sending satisfaction surveys to a random sampling of 30 healthcare providers (physicians, nurses, and allied health professionals) throughout the hospital who consulted palliative care on a quarterly basis, we extracted neonatal staff members’ responses from this survey to attain these scores.

## RESULTS

### Primary Outcome Measure

**Fig. 2.**
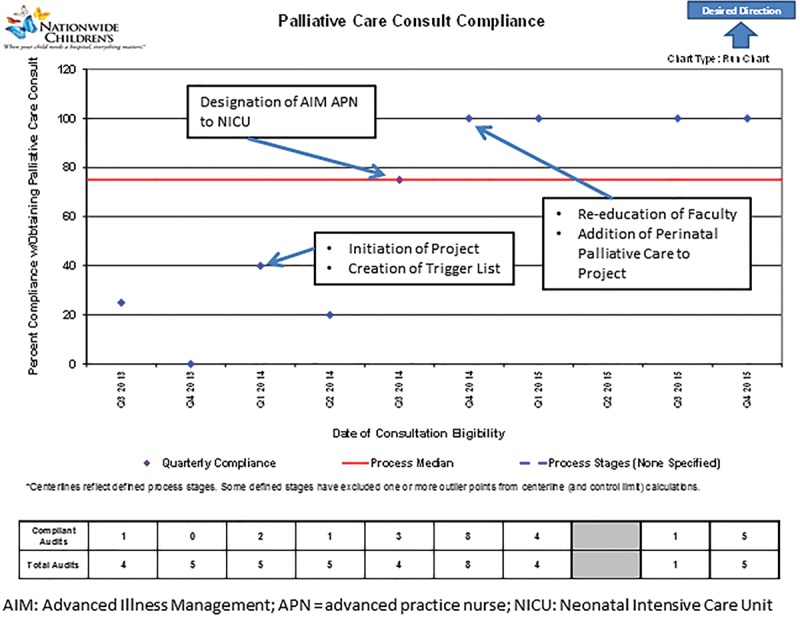
Run chart for pediatric palliative care consult compliance quality improvement project.

The specific aim of the project was to increase PPC consultations for those infants identified on the revised diagnosis “trigger” list from 25% to 80% and to maintain this increase for 6 months. We achieved 100% compliance within 12 months (Fig. 2). The following interventions were most impactful: (1) creation of an NICU severe diagnosis “trigger” list; (2) identification of a primary PPC provider for the NICU; and (3) expansion of the perinatal palliative care program.

The creation and refinement of a PPC eligible trigger list resulted in 5 diagnoses that consistently resulted in high-acuity patients at high risk for mortality warranting PPC consultation. Over the 2-year period, 21 postnatal patients met trigger list eligibility. Thirteen received PPC consultation.

Another strategy was the addition of a dedicated PPC team member in the NICU. In quarter 3 of 2014, the PPC team selected an advanced practice nurse to focus on the NICU. Before this intervention, trigger-driven consultation rates were at 20%. At the end of quarter 3 and initiation of this intervention, compliance rose to 75%.

**Fig. 3.**
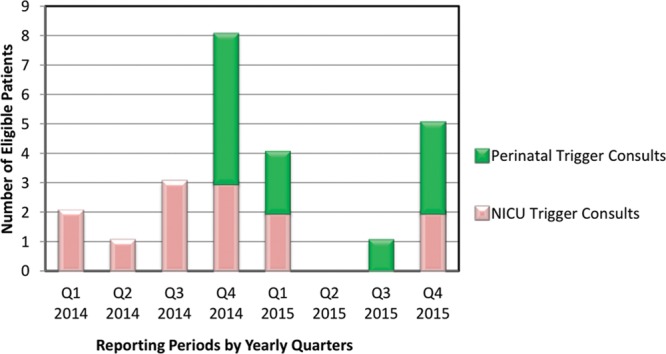
Completion of NICU and perinatal trigger list eligible consultations.

The final intervention was the revamping of the perinatal palliative care program. Compliance with the severe diagnosis trigger list increased from 75% to 100% by the fourth quarter of 2014. While there was a decline in the overall number of eligible NICU patients from Q1 to Q3 2015 (zero in 6 months), the completed perinatal palliative consults for infants with trigger eligible diagnoses increased from baseline from Q3 2014 to Q4 2015 (Fig. 3). Over the 2-year project, 11 prenatal patients were eligible for PPC consultation by trigger list diagnosis and the family was seen by the perinatal palliative care program before delivery. Within these eligible perinatal consultations, 4 were ultimately intrauterine fetal demises, 3 were neonatal deaths at their birth hospitals based on care plan to not transfer to NICU nor to go home with hospice, 1 went home with hospice, 2 were lost to follow-up, and 1 transferred to the NICU and then seen by the inpatient palliative care team. Counting this patient as twice eligible for consultation, the total number of prenatal and postnatal patients eligible for PPC consultation during this project period was 31 with 24 patients receiving prenatal, postnatal, or both PPC consultation.

### Process Measure Results

The project’s process measure was the time from hospitalization to PPC consultation for eligible patients using the severe diagnosis trigger list. The time for consultation over the course of the project had no meaningful change (**Supplemental Digital Content 1, available at** http://links.lww.com/PQ9/A62).

### Balancing Measure Results

The balancing measure during this project was to review NICU staff satisfaction for those interacting with the PPC team. **Supplemental Digital Content 2 (available at** http://links.lww.com/PQ9/A63) reveals both the questions asked and the responses of the staff survey. Overall, the 6 queried staff, which included physician, chaplain, social work, and nursing feedback, reported satisfaction with the PPC team, its psychosocial and spiritual support, and a willingness to reconsult palliative care in the future.

## DISCUSSION

In this report, we describe the improvement in the rate of PPC consultation in a select population of neonates whose diagnoses were life-threatening and required complex medical decision making. This QI project requested attending neonatologists to obtain PPC consultation for 5 identified high-risk diagnoses. The PPC consultation rate for this population of patients increased from 25% at baseline to 100% and maintained this level of consultation for 1 year. Simultaneously, staff satisfaction with the PPC services remained high.

The most impactful interventions were (1) engagement of the neonatology faculty in the refinement of the list of potential diagnoses to a final 5 diagnoses; (2) dedication of a PPC staff member to the NICU, and (3) expansion of the perinatal palliative care program.

One significant intervention was establishing trigger criteria for initiating PPC. This proven technique among adults for certain disease groups and settings^5,6^ has also increased PPC’s presence in our pediatric intensive care unit.^7^ Some palliative care programs utilize diagnosis-based trigger lists to initiate palliative care consultations, but other programs have expressed concerns that automated consultation may misidentify a patient’s acuity and palliative needs. Specifically, triggers could cause unnecessary palliative consults on some patients and miss eligible patients who lack a trigger diagnosis. Premature or unnecessary consults are particularly concerning for a PPC team, which is often thinly staffed and relies upon accurate patient identification to optimize PPC access.

To allay these concerns while working toward the project’s global aim of increasing PPC’s presence in the NICU, we placed great scrutiny on the trigger list itself. The QI group reviewed these data on a monthly basis (**Supplement Digital Content 3, available at** http://links.lww.com/PQ9/A64) and after 5 monthly cycles (April 1, 2014, to September 1, 2014) noted that some consults with the same diagnostic codes lacked a level of acuity to warrant a PPC consult. For example, ICD-9 codes for myelomeningocele were often utilized when the work-up for myelomeningocele was underway but rarely did the work-up ultimately reveal myelomeningocele. Additionally, in times when myelomeningocele was diagnosed, it was often an isolated diagnosis with a degree of decision making that the NICU staff could solely support. This observation is in contradiction to the QI team’s hypothesis that the diagnosis would travel with a multitude of diagnoses with high medical decision-making needs warranting PPC consultation and thus its inclusion in the trigger list. As this list was to represent a list of diagnoses that automatically led to PPC consultation, the need to accurately identify high medical complexity was paramount. Thus, we modified the trigger list to 5 diagnoses (Table 2), which consistently resulted in inpatient death or home-based palliative/hospice eligibility upon discharge to better capture this level of need.

One significant shortcoming of the project was the infrequent eligibility of patients. By modifying the trigger list to 5 critical diagnoses, the project excluded infants with other potentially life-limiting diagnoses who died in the NICU. Specifically, the extremely premature infant who died soon after delivery was purposefully excluded from trigger list criteria as were the patients with multiorgan failure, metabolic disorder, and/or genetic disorder despite the latter 2 diagnoses comprising the national majority of PPC’s patient population.^8^ The global aim of the project was to increase PPC presence in the NICU. This project’s working group quickly recognized that accepting an incomplete trigger list that the NICU faculty fully supported was the best option to reach the global aim. With this approach, total perinatal and NICU PPC consults (regardless of diagnosis) grew each year since the inception of this project (**Supplemental Digital Content 4, available at** http://links.lww.com/PQ9/A65). This subsequent growth was organic and not driven by subsequent QI work but underscores the ripple effect possible from this type of QI work.

Finally, the increased perinatal palliative care consultations impacted project results as this trend came with a noticeable fall in trigger eligible patients in our NICU. This result could have occurred because pregnant families of infants with trigger list diagnoses who received a perinatal palliative care consultation during this study period often chose to minimize interventions for their baby and did not transfer to our NICU. This led to a decreased number of eligible patients in our NICU as only one of the perinatal consults for a trigger eligible patient was transferred to NCH after birth. While this effect was not a primary outcome goal of the project, it was an interesting trend that will inform future QI projects at our organization as it suggests early palliative care contact could impact parental decision making in the perinatal population.

## CONCLUSIONS

This QI study demonstrates a successful process to create, refine, and implement a severe diagnosis trigger list for neonates that would benefit from PPC consultation in the NICU. We increased PPC consultation rates for families of infants with severe diagnoses in the NICU from 25% to 100% compliance within 6 months and sustained for 1 year. Simultaneously, the project supported the growth of the perinatal palliative care program. We also showed improvement in our global aim to improve PPC’s presence in the NICU. Through such measures, we hope to advance our program’s mission of serving families of children with life-threatening conditions within the NICU and beyond.

## DISCLOSURE

The authors have no financial interest to declare in relation to the content of this article.

## Supplementary Material
