# Supplementary material for: Trigger Criteria to Increase Appropriate Palliative Care Consultation in the Neonatal Intensive Care Unit
Source: Pediatr Qual Saf. 2019 Feb 7;4(1):e129. doi: 10.1097/pq9.0000000000000129 (PMC6426490; doi:10.1097/pq9.0000000000000129)
Supplement: Supplementary file 1 [file pqs-4-e129-s001.docx]

**Supplement 1**: Time from Admission to Hospital to AIM Consultation in Trigger List Eligible Patients

| **Quarter** | **Number of AIM consults** | **Mean Time to Consultation (days)** |
| --- | --- | --- |
| Q2 2014 | 1 | 7 |
| Q3 2014 | 3 | 3 |
| Q4 2014 | 3 | 2 |
| Q1 2015 | 2 | 7 |
| Q2 2015 | 0 | 0 |
| Q3 2015 | 0 | 0 |
| Q4 2015 | 2 | 4 |
