# Supplementary material for: Trigger Criteria to Increase Appropriate Palliative Care Consultation in the Neonatal Intensive Care Unit
Source: Pediatr Qual Saf. 2019 Feb 7;4(1):e129. doi: 10.1097/pq9.0000000000000129 (PMC6426490; doi:10.1097/pq9.0000000000000129)
Supplement: Supplementary file 2 [file pqs-4-e129-s002.docx]

Supplement 2: NICU Staff Satisfaction Survey

1. In general, how easy was it to refer a patient?

| **Not easy at all** | **Somewhat difficult** | **Average** | **Easier than Expected** | **Extremely Easy** | **I Don’t Know** |
| --- | --- | --- | --- | --- | --- |
| 0 | 0 | 0 | 0 | 66.67% | 33.33% |

1. How promptly were your request for consults responded to?

| **Not promptly at all** | **Slower than Average** | **Average** | **Faster than Average** | **Extremely Prompt** | **I Don’t Know** |
| --- | --- | --- | --- | --- | --- |
| 0 | 0 | 0 | 16.7% | 66.7% | 16.7% |

1. Please indicate how helpful the AIM team was in assisting with each topic:

|  | **Not Helpful At All** | **Somewhat Helpful** | **Helpful** | **Extremely Helpful** | **Not Applicable** |
| --- | --- | --- | --- | --- | --- |
| **Emotional Support for You** | | | | | |
|  | 0 | 0 | 50% | 50% | 0 |
| **Emotional Support for the Healthcare Team** | | | | | |
|  | 0 | 0 | 50% | 50% | 0 |
| **End of Life Decision Making** | | | | | |
|  | 0 | 0 | 83.3% | 16.7% | 0 |
| **Family Distress/Anticipatory Grief** | | | | | |
|  | 0 | 0 | 33.3% | 66.67% | 0 |
| **Homecare Planning/Hospice Referral** | | | | | |
|  | 0 | 0 | 16.7% | 66.7% | 16.7% |
| **Psychological Measures** | | | | | |
|  | 0 | 0 | 83.3% | 16.7% | 0 |
| **Spiritual Support** | | | | | |
|  | 0 | 50% | 33.3% | 0 | 16.7% |

1. Overall, how helpful was the AIM team to you and the whole healthcare team?

| **Not Helpful at All** | **Somewhat Helpful** | **Helpful** | **Extremely Helpful** |
| --- | --- | --- | --- |
| 0 | 0 | 50% | 50% |

1. Overall, how helpful was the AIM team to the family?

| **Not Helpful at All** | **Somewhat Helpful** | **Helpful** | **Extremely Helpful** |
| --- | --- | --- | --- |
| 0 | 0 | 50% | 50% |

1. Did you perceive barriers to AIM team's involvement?

| **Availability of AIM Team** | **Availability of Family at Bedside** | **Family Hesitancy** | **Provider Hesitancy** | **No Barriers** |
| --- | --- | --- | --- | --- |
| 0 | 0 | 33.3% | 33.3% | 33.3% |

1. Overall, how satisfied are you with your experience(s) with the AIM team?

| **Not Satisfied at All** | **Somewhat Satisfied** | **Satisfied** | **Extremely Satisfied** |
| --- | --- | --- | --- |
| 0 | 0 | 33.3% | 66.7% |

1. Would you refer to the AIM team again?

| **No** | **Yes** |
| --- | --- |
| 0 | 100% |
