# Supplementary material for: Trigger Criteria to Increase Appropriate Palliative Care Consultation in the Neonatal Intensive Care Unit
Source: Pediatr Qual Saf. 2019 Feb 7;4(1):e129. doi: 10.1097/pq9.0000000000000129 (PMC6426490; doi:10.1097/pq9.0000000000000129)
Supplement: Supplementary file 3 [file pqs-4-e129-s003.docx]

**Supplement 3**: Initial Trigger List Eligibility and Compliance Rates in the Neonatal Intensive Care Unit (NICU) with Discharge Outcomes.

| **Cycle Month** | **# Trigger eligible patients** | **# Patients w/ AIM consult** | **# Missed Consults** | **# Patients that died** | **# Patients home hospice or PPC eligible** | **# Patients not PPC eligible at discharge** |
| --- | --- | --- | --- | --- | --- | --- |
| **April** | 2 | 1 | 1 | 1 | 0 | 1 |
| **May** | 3 | 0 | 3 | 0 | 0 | 3 |
| **June** | 0 | 0 | 0 | 0 | 0 | 0 |
| **Q2 2014 Sums** | **5** | **1** | **4** | **1** | **0** | **4** |
| **July** | 1 | 1 | 0 | 1 | 1 | 0 |
| **August** | 2 | 1 | 1 | 0 | 0 | 2 |
| **September** | 1 | 1 | 0 | 1 | 0 | 0 |
| **Q3 2014 Sums** | **4** | **3** | **1** | **2** | **0** | **2** |
| **October** | 1 | 1 | 0 | 1 | 0 | 0 |
| **November** | 1 | 1 | 0 | 0 | 1 | 0 |
| **December** | 1 | 1 | 0 | 1 | 0 | 0 |
| **Q4 2014 Sums** | **3** | **3** | **0** | **2** | **1** | **0** |
| **January** | 1 | 1 | 0 | 0 | 0 | 1 |
| **February** | 1 | 1 | 0 | 1 | 0 | 0 |
| **March** | 0 | 0 | 0 | 0 | 0 | 0 |
| **Q1 2015 Sums** | **2** | **2** | **0** | **1** | **0** | **1** |
| **April** | 0 | 0 | 0 | 0 | 0 | 0 |
| **May** | 0 | 0 | 0 | 0 | 0 | 0 |
| **June** | 0 | 0 | 0 | 0 | 0 | 0 |
| **Q2 2015 Sums** | **0** | **0** | **0** | **0** | **0** | **0** |
| **July** | 0 | 0 | 0 | 0 | 0 | 0 |
| **August** | 0 | 0 | 0 | 0 | 0 | 0 |
| **September** | 0 | 0 | 0 | 0 | 0 | 0 |
| **Q3 2015 Sums** | **0** | **0** | **0** | **0** | **0** | **0** |
| **October** | 1 | 1 | 0 | 0 | 1 | 0 |
| **November** | 1 | 1 | 0 | 1 | 0 | 0 |
| **December** | 0 | 0 | 0 | 0 | 0 | 0 |
| **Q4 2015 Sums** | **2** | **2** | **0** | **1** | **1** | **0** |
