# Supplementary material for: Trigger Criteria to Increase Appropriate Palliative Care Consultation in the Neonatal Intensive Care Unit
Source: Pediatr Qual Saf. 2019 Feb 7;4(1):e129. doi: 10.1097/pq9.0000000000000129 (PMC6426490; doi:10.1097/pq9.0000000000000129)
Supplement: Supplementary file 4 [file pqs-4-e129-s004.docx]

Supplement 4: Total Count Trigger and Non-Trigger Perinatal and NICU Palliative Care Consultations

| **Year** | **Prenatal PC Consult** | **NICU PC Consult** | **Total PC Consults** |
| --- | --- | --- | --- |
| 2013 | 0 | 27 | 27 |
| 2014 | 5 | 38 | 43 |
| 2015 | 14 | 44 | 58 |
| 2016 | 16 | 56 | 72 |
| 2017 | 24 | 52 | 76 |
